# Prevalence of electronic screening for sepsis in National Health Service acute hospitals in England

**DOI:** 10.1136/bmjhci-2023-100743

**Published:** 2023-05-11

**Authors:** Kate Honeyford, Amen-Patrick Nwosu, Runa Lazzarino, Anne Kinderlerer, John Welch, Andrew J Brent, Graham Cooke, Peter Ghazal, Shashank Patil, DiAlS Co-investigators, Ceire E Costelloe

**Affiliations:** 1 Team Health Informatics, Institute of Cancer Research, London, UK; 2 MRC Centre for Global Infectious Disease Analysis, Jameel Institute, School of Public Health, Imperial College London, London, UK; 3 Nuffield Department of Primary Care and Health Sciences, University of Oxford, Oxford, UK; 4 Imperial College Healthcare NHS Trust, London, UK; 5 Critical Care Department, University College Hospital, London, UK; 6 Oxford University Hospitals NHS Foundation Trust, Oxford, UK; 7 Nuffield Department of Medicine, University of Oxford, Oxford, UK; 8 Department of Infectious Disease, Imperial College, London, UK; 9 National Institute for Health Research Imperial Biomedical Research Centre, London, UK; 10 Systems Immunity Research Institute, School of Medicine, Cardiff University, Cardiff, UK; 11 Emergency Department, Chelsea and Westminster Healthcare NHS Trust, London, UK; 12 Health Informatics Team, Royal Marsden NHS Foundation Trust, London, UK

**Keywords:** electronic health records, decision support systems, clinical, medical records systems, computerized, delivery of health care

## Abstract

Sepsis is a worldwide public health problem. Rapid identification is associated with improved patient outcomes—if followed by timely appropriate treatment.

**Objectives:**

Describe digital sepsis alerts (DSAs) in use in English National Health Service (NHS) acute hospitals.

**Methods:**

A Freedom of Information request surveyed acute NHS Trusts on their adoption of electronic patient records (EPRs) and DSAs.

**Results:**

Of the 99 Trusts that responded, 84 had an EPR. Over 20 different EPR system providers were identified as operational in England. The most common providers were Cerner (21%). System C, Dedalus and Allscripts Sunrise were also relatively common (13%, 10% and 7%, respectively). 70% of NHS Trusts with an EPR responded that they had a DSA; most of these use the National Early Warning Score (NEWS2). There was evidence that the EPR provider was related to the DSA algorithm. We found no evidence that Trusts were using EPRs to introduce data driven algorithms or DSAs able to include, for example, pre-existing conditions that may be known to increase risk.

Not all Trusts were willing or able to provide details of their EPR or the underlying algorithm.

**Discussion:**

The majority of NHS Trusts use an EPR of some kind; many use a NEWS2-based DSA in keeping with national guidelines.

**Conclusion:**

Many English NHS Trusts use DSAs; even those using similar triggers vary and many recreate paper systems. Despite the proliferation of machine learning algorithms being developed to support early detection of sepsis, there is little evidence that these are being used to improve personalised sepsis detection.

**WHAT IS ALREADY KNOWN ON THIS TOPIC**

- Digital alerts are being introduced into hospital systems in England as they switch to electronic patient records (EPRs). Little is known about the presence of digital sepsis alerts in these hospitals or the accuracy of the underlying algorithms.

**WHAT THIS STUDY ADDS**

- The majority of hospitals with EPRs use digital sepsis alerts, with National Early Warning Score 2 being the most common algorithm to detect all-cause deterioration including sepsis. The algorithm in use is influenced by the EPR contracted by the Trust.

**HOW THIS STUDY MIGHT AFFECT RESEARCH, PRACTICE OR POLICY**

- Detailed patient data within EPRs is not currently exploited to improve digital sepsis alerts in hospitals. We recommend that NHS organisations are open about the digital tools in use and their effectiveness rigorously evaluated.

## Introduction

Sepsis is a worldwide public health problem, with a recent report estimating a 11 million global death toll in 1 year alone. Early diagnosis and management is crucial to improve patient outcomes,^1 2^ with inconsistent recognition and management of sepsis being repeatedly highlighted as a safety concern in hospital service/quality of care audits.^3^

These related issues currently make early sepsis recognition more challenging: interindividual heterogeneity in the underlying aetiology and clinical phenotype; inconsistency in the implementation of a consensus clinical definition; and most critically, the lack of a reliable test for sepsis.^4^

Screening for sepsis is widely implemented across countries, and is essential for prompt treatment and optimal outcomes.^5^ Latest international guidelines recommend that all hospitals and healthcare systems adopt sepsis performance improvement programmes, which include the use of screening tools to promptly identify sepsis.^1 6^ However, compliance with these guidelines is not universal, and implementation is an ongoing challenge.^7^

**Figure 1:**
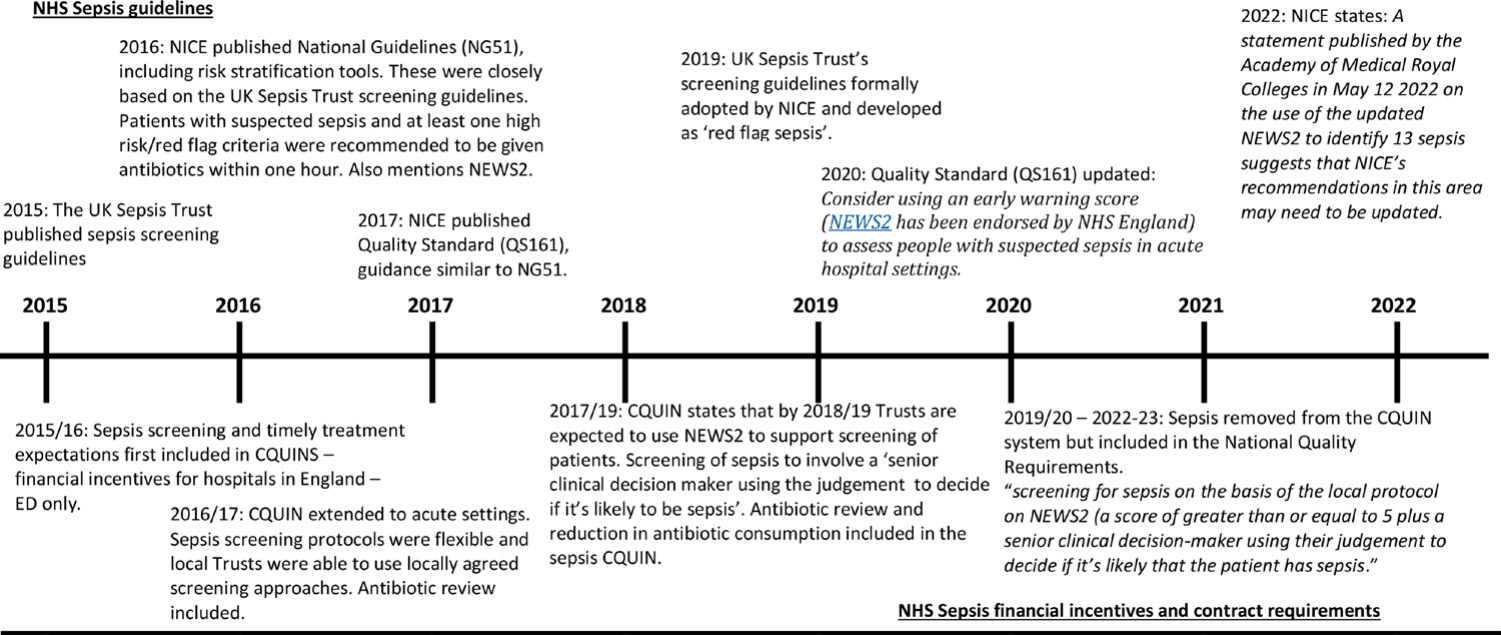
Timeline showing the development of sepsis guidelines and incentives in the NHS in England. NEWS2, National Early Warning Score version details of NICE guidelines and quality Standards are available at: www.nice.org.uk/guidance/conditions-and-diseases/infections/sepsis, details of CQUINs are available at: www.england.nhs.uk/nhs-standard-contract/cquin/, details of NHS standard contract are available at: www.england.nhs.uk/nhs-standard-contract/previous-nhs-standard-contracts/. CQC, Care Quality Commission; CQUIN, Commissioning and Quality Innovation; ED, emergency department.; NICE, National Institute of Clinical Excellence; NHS, National Health Service

Currently, hospitals in England are required to screen both emergency department (ED) patients and inpatients for sepsis ‘where appropriate’ and there have been associated financial incentives towards this.^8^ Recent guidelines are summarised in figure 1. To date, none of these guidelines considers the use of electronic tools to aid screening, or their potential advantages and disadvantages.

Despite their absence from current guidelines, electronic screening tools for sepsis have been in use in English hospitals for over 5 years. Previous work from our group showed that the introduction of a digital sepsis screening tool and accompanying alert was associated with reduction in risk of mortality, and an increase in timely treatment with antibiotics.^9^ Individual Trusts have identified improvements in patient outcomes including reductions in septic shock in under 45s from 60% to 7.7%,^10^ 70% increase in patients diagnosed with sepsis receiving antibiotics within the target time frame, and 64 potential lives saved 1 year.^11^ However, these claims have not been peer reviewed, or adjusted for underlying trends and casemix.

Currently, most electronic screening tools for sepsis available in England are rule based, track and trigger (T&T) systems, that is, systems which rely on periodic observation of selected physiological signs with predetermined criteria for escalating care.^12^ The most commonly available tools include systemic inflammatory response syndrome (SIRS) criteria, quick Sepsis-related Organ Failure Assessment (qSOFA), modified Early Warning Scores and, in the UK, National Early Warning Score (NEWS)2.^6^ SIRS and qSOFA were initially developed as diagnostic tools for sepsis, but are now commonly used for highlighting patients at risk of poor outcomes from sepsis (details are shown in table 1).^4^ These tools often have high sensitivity, but low specificity.^6^ The criteria of these tools are applicable to adults and are not directly appropriate for neonates, children or maternity patients; consequently, this paper focuses on digital sepsis tools for use in adults.

**Table 1:** Summary of key criteria used in sepsis detection algorithms

Clinical observation	qSOFA*	NEWS2 Consider infection			Red Flag NEWS3+possible infection One from lists below		SIRS*	St John sepsis algorithm
		1	2	3	Red	Amber		
Respiration Rate	³22		21–24	≥25	25	21–24	>20	>21
		9–11		<8				
O_2_ Saturation	–	94–95	92–93	<91	<91	–	–	–
Supplemental O_2_	–		Yes		>92 with supp O_2_	–	–	–
Temperature, °C	–	35.1–36.1		<35	–	<36	<36.0 or >38.0	<36.0 or >38.3
		38.1–39.0	³39.1					
Systolic blood pressure	<100	101–110	91–100	<90	<90	91–100		<90
				>220				
Heart rate	–	41–50		<40	>130	91–130	>90	>95
		91–110	111–130	³131				
Level of consciousness	GCS<13 or AMS			ACVPU	ACVPU	Concern	–	–
Lactate	–	–	–	–	³2.0 mmol	–	–	³2.0 mmol
Glucose mg/dL	–	–	–	–	–	–	–	>140 and <200
Fluid balance	–	–	–	–	No urine 18+ hours or <0.5 mL/kg/hour	No urine 12–18 hours	–	–
White cell count	–	–	–	–	–	–	>12 k or <4 k or band >10%	>12 k or <4 k or band >10%
Creatinine mg/dL (μmol/L)	–	–	–	–	–	–	–	Increase of 0.5 over 72 hours
Bilirubin mg/dL	–	–	–	–	–	–	–	<2.0 and < 10.0

*SIRS and qSOFA have previously been included in sepsis diagnostic criteria; in this report, we focus on the use of these tools as prediction/alerting algorithms.

ACVPU, alert, confusion, voice, pain, unresponsive; AMS, altered mental status; GCS, Glasgow Coma Score; NEWS, National Early Warning Score; qSOFA, quick Sepsis-related Organ Failure Assessment; SIRS, systemic inflammatory response syndrome.

Current UK adult sepsis guidelines recommend using NEWS2 (see figure 1) to identify patients at risk of deterioration and then involve a senior clinical decision maker to determine if sepsis is driving the deterioration. This is a simple approach that can easily be linked to electronic systems. None of these algorithms, including NEWS2, makes use of the granular nature of electronic patient records (EPRs); for example, pre-existing conditions and treatments or deviations in vital signs from the normal for an individual patient. This is despite published studies highlighting the benefits and high predictive performance of algorithms based on machine learning approaches which can factor in more detailed patient information.^13^ These studies were not conducted in hospital settings, hence evidence of positive results in hospital settings is still limited.^14^ Indeed, few digital sepsis alerts (DSAs) available to hospitals have been evaluated in terms of patient benefit as opposed to predictive accuracy.

As the UK National Health Service (NHS) seeks to become paperless and embraces digital technology, the incorporation of digital alerts embedded within the EPR is an attractive option to aid clinical decision-making, and has the potential to increase the quality, efficiency and cost-effectiveness of sepsis care. However, little is known about the digital alerts currently in use or the rationale for their inclusion in healthcare systems. In the case of sepsis, there is some emerging evidence of the effectiveness of these tools, but there are no validated digital tools available to NHS Trusts which have been shown to be effective in improving patient outcomes in a range of settings, nor has there been a recent comprehensive review of the algorithms in use.

In this paper, we describe DSAs, based on English NHS Trusts responses to Freedom of Information (FOI) request.

## Methods

Working with a group of close collaborators identified to be part of a wider project (see box 1 for further details), we identified key aspects of algorithms in use in five NHS hospitals to inform our further work. We used an FoI request to survey all hospitals and used internet searching to gather additional information.

Box 1 **DiAlS—Digital Alerting for Sepsis**

The DiAlS study is investigating the impact of DSAs on patient outcomes and staff activity in six NHS hospital Trusts across England and Wales.

The implementation of digital alerts in hospitals is a complex health intervention. Therefore, we are using a mixed-methods approach to ensure understanding of the relationship between inherent aspects of the alerts, such as the underlying algorithm and the method of clinician notification. Using appropriate qualitative and quantitative methods, based on the analysis of natural experiments, we will evaluate the implementation of alerts across six NHS Trusts, most of which have adopted distinctive digital alerts.

Outcomes will include in-hospital mortality within 30 days, transfers to the intensive care unit, length of stay and administration of intravenous antibiotics. We will also consider unintended consequences related to unnecessary and inappropriate use of antibiotics.

DiAlS is funded by National Institute for Health and Care Research (NIHR) Health and Social Care Delivery Research (HS&DR) and is working in collaboration with NIHR-Health Informatics Collaborative.

DSAs, digital sepsis alerts.

The FoI request was submitted to all acute NHS hospital Trusts in England that have an ED (with the exception of the five NHS hospitals in the pilot work), to collect information on EPRs; electronic sepsis screening tools and the underlying algorithms they use; the association between the underlying algorithm and the alerts to clinicians; the timing of introduction of the electronic screening tool in the hospital; and which staff groups see and respond to alerts. We did not give a definition of EPR in our request which enabled trusts to respond how they deemed most appropriate. The FoI request is available in online supplemental appendix 1.

10.1136/bmjhci-2023-100743.supp1 Supplementary data

The results of the FoI were screened by two authors (KH and A-PN). Where there was ambiguity in the response by the trust, for example, if the response indicated that there was no DSA but details of the algorithm and process were supplied, we discussed the response and reached a consensus approach.

## Results

FOI of requests were sent to 120 Acute NHS Trusts which had EDs. Responses were received from 94 NHS Trusts. Additional information was gathered from the Digital Alerts for Sepsis (DiAlS) clinical team and from five NHS Trusts participating in DiAlS (see box 1). Of the 99 Trusts for which information was available, 14 (14%) responded that they did not have an electronic health record or EPR. Eighty-four (85%) Trusts responded that they had an EPR. The most common single provider was Cerner (18 Trusts, 21%). System C, Dedalus and Allscripts Sunrise were also relatively common (13%, 7% and 10%, respectively). Four Trusts used Epic and two used in-house systems. Over one-fifth of Trusts (22%) identified a mix of companies providing their EPRs, with EDs and inpatient wards sometimes using different systems, and some identifying various patient administration systems. Further details are provided in table 2. One Trust refused to provide information on their provider citing potential cyberattacks as justification.

**Table 2:** EPR providers, digital sepsis alerts and associated algorithms

	All EPRs		NEWS2					qSOFA alone	Red Flag alone	SIRS alone
		Sepsis alerts	Alone	& qSOFA	& Red Flag	& sepsis screen	& SIRS			
**Total**	**84**	**59**	**22**	**9**	**6**	**8**	**1**	**3**	**8**	**2**
Mix	19 (23%)	14 (24%)	4	3	2	3			2	
Cerner	18 (21%)	15 (27%)	6		2	–	1		4	2
System C	11 (13%)	8 (14%)	1	5		–		1		
Allscripts Sunrise	6 (7%)	3 (5%)	1			1		1	1	
Dedalus	8 (10%)	3 (5%)	1		1	1				
In-house	2 (5%)	1 (2%)	–			1				
Epic	4 (5%)	3 (5%)	2			1				
Other	14 (17%)	8 (15%)	5	1	1	–		1	1	
Missing	2 (2%)	–	1			1				

Percentages are column percentages, showing the proportion of each algorithm associated with each of the main EPR provider.

EPR, electronic patient record; qSOFA, quick Sepsis-related Organ Failure Assessment; SIRS, systemic inflammatory response syndrome.

DSAs were reported to be in use in 59 of the 85 digital Trusts (69%). Systems based on NEWS2 were the most used across all systems (46 Trusts (78%)). Of these, 29 used a combined approach, an aggregate score of 5 or above or a single parameter of 3 or above, compared with 21 which either specified that they use a score of 5 or above or did not specify. Within Trusts which use NEWS2, 24 (52%) use an additional screening tool; these include Red Flag Sepsis^15^ and qSOFA.^4^ A further eight NEWS2 Trusts use an additional screening tool, such as asking for an indication of infection, and one uses SIRS criteria. Some Trusts indicated that their digital system prompts a question or a series of questions about the possibility of sepsis, but there was no evidence that the responses to these were precompleted by the electronic system, despite some of this information being available within the EPR. Eight Trusts responded that they used Red Flag criteria as a stand-alone assessment, while three used qSOFA and two SIRS.

Five Trusts did not give sufficient information to determine the algorithm behind the sepsis alert. An additional four were unwilling to provide information on the algorithm, and three trusts use bespoke systems which were a modification of NEWS2 or Red Flag Sepsis.

We saw some patterns in the algorithm used and the EPR provider. These are summarised in table 2. The EPR provider for all Trusts which use a combination of NEWS2 and qSOFA is system C and for those that use an SIRS-based system it is Cerner. Cerner was also a common provider for Trusts using Red Flag Sepsis alone or in combination with NEWS2.

### Willingness to disclose information

As identified above, not all NHS Trusts were happy to disclose information and some Trusts were unable to provide information. Some of these responses are provided in box 2.

Box 2 **Example of responses from trusts which were unable or unwilling to provide information on EPR provision or digital sepsis alert algorithms.**

‘The Trust considers this question to be exempt from disclosure in accordance with section 43.2 of the Freedom of Information Act as to release this information would, or would be likely to, prejudice the commercial interests of the supplier.’

‘Care Flow Vitals Clinical uses qSOFA scoring for detection of patients at risk of sepsis. The exact algorithm is not known by the Trust.’

‘N/K’.

‘“Unable to provide as this is managed by the supplier’.

‘In view of cybersecurity attacks on organisations, the Trust considers that public release of this information could put the Trust’s system and information contained on that system at risk. Accordingly the Trust considers, therefore, that section 31(1) of the Freedom of Information Act 2000 applies. As this is a qualified exemption, the Trust has applied the public interest test as required and deems that, on balance and for the reason stated above, the public interest lies in not disclosing this information.’

‘Unable to provide as the algorithms are part of a third party system and proprietary knowledge’.

EPR, electronic patient record.

## Discussion

The majority of Trusts responding reported having an EPR and over 20 different providers were identified as operational in NHS Trusts in England. Three-quarters of digital Trusts responded that they had a DSA and most these use NEWS2 as part of their sepsis alerting system. This is the approach included in the NHS National Standard Contract.^16^

There is evidence that the provider of the EPR in use in the hospital is associated with the underlying sepsis algorithm in use in the Trust. For example, SIRS-based alerts are only found in Trusts where the provider is Cerner and qSOFA is part of the System C sepsis alert system.

Given NEWS2 is the nationally recommended system for identifying patients who need to be screened for sepsis it is not surprising that NEWS2 is the most commonly used system, usually with a threshold of 5 as the trigger for review and consideration of sepsis. In addition, many Trusts include a score of 3 in any single parameter, despite national guidance moving away from this approach as a trigger for significant escalation as it is a poor predictor of risk.^17^ This may be a legacy of the overlap between indicators in Red Flag Sepsis and NEWS2.

There was no evidence that Trusts’ digitisation of patient health records was associated with the introduction of more complex algorithms, either data-driven, machine learning-based algorithms or algorithms which were able to include pre-existing conditions or patient information.

Our review of the EPR systems in use in English Trusts are in line with those found by Warren *et al*.^18^ In their study of NHS Trusts (2017–2018), 23% of Trusts reported having no electronic system, suggesting an increase in adoption of electronic systems since 2018. Cerner was the most commonly reported provider (18%), then DXC (13%) and System C (11%). DXC were the providers of the Lorenzo EPR in 2017, but were bought out by Dedalus, an Italian-based provider, in April 2021,^19^ which was still a common provider in our survey (10%). We found a higher proportion with a mixed system than Warren *et al*, which may reflect a less precise definition of EPR in our study or changes over time.

In 2007, the NIHR reported that ‘T&T systems were in widespread use in NHS acute hospitals’; it is therefore no surprise that we have found that the majority of digital trusts are using digital T&T systems as key components of their DSAs.^20^ In addition, Trusts are expected to use NEWS2 as a screening system for deteriorating patients. Advantages of T&T systems include the ability to monitor large numbers of patients without ‘a large increase in workload’, and digital enhancement of these systems has clear advantages, for example, one study showed errors in pen-and-paper T&T systems, with errors in 29% calculated scores reviewed (n=84), half of these led to incorrect clinical action.^12^ Although NEWS2 is a relatively simple system, there is an obvious advantage in clinical data being aggregated automatically and removing the need for busy clinical staff determining and totalling the ‘points’ value of each observation.

In addition to NEWS2, qSOFA and SIRS were relatively commonly used, with adoption associated with the EPR provider. qSOFA was also used in addition to the expected NEWS2. It is possible to trigger a qSOFA score of 2 or more without aggregating to a score of 5 or more in NEWS2, but there is no evidence that using the two combined leads to improved specificity.^21^

SIRS-based systems were only in use in Trusts where Cerner was the EPR provider. SIRS is not now currently considered a useful way of defining sepsis, but may be useful in predicting poor outcomes for patients.^9 22^ SIRS-based algorithms do make use of more detailed information contained within patient records, for example, recent lactate and bilirubin levels which reflect organ dysfunction. In addition, the Cerner-based algorithm can be set different thresholds for patients with diabetes or undergoing dialysis, the only system which automatically considers wider information about the patient. However, most studies suggest that SIRS has limited utility in accurately identifying sepsis^21^ or predicting mortality in patients.^23^

NEWS2 and qSOFA were designed to be easily performed at the bedside and qSOFA was developed using a parsimonious model to achieve a ‘simple scoring system with the fewest number of variables associated with the greatest predictive ability’.^24^ Although this approach makes sense in low-resource settings without EPRs, it does not take advantage of the available granular patient data. This includes information from the current visit, previous contacts with the hospital and potentially information from recent primary care appointments.

In this paper, we have not examined the potential benefits or harms of DSAs; a systematic review^25^ did not find a reduction in mortality, in contrast to Honeyford *et al*.^9^ Studies have shown improvements in achievement of process measures. In different parts of the hospital, alerts’ potential to improve patient outcomes varies; in modern EDs, there is often continuous electronic monitoring. In highly resourced EDs, unwell patients are usually reviewed early by a senior clinician, hence there may be limited value of an alert system. Where staff are under increased pressure, alerts may be more important.^25^ This contrasting evidence emphasises the need for robust evidence to determine the most appropriate DSA.

A minority of Trusts were unwilling or unable to give detailed information about their EPR or the underlying algorithm used for their sepsis alert. This is an important aspect of the introduction of digital alerts in hospitals in England/UK. Currently, there is no clear approval process for digital alerts, and, hence, no necessity for hospitals to use ‘approved’ digital alerts. Two high-profile alerts have recently been identified at best as having no utility, at worst, causing patient harm.^14 26^

Initially, it was hypothesised that Trusts who responded that they had an EPR would be paperless or heavily paper reduced. However, responses to the question indicate some Trusts are combining electronic and paper systems; we were, therefore, unable to determine how many Trusts are paper reduced/less. We had similar challenges in determining the level of ‘digital’ in the sepsis alert. While some Trusts answered ‘yes’ to DSAs, examination of the details provided suggested that the alert relied on paper. The UK Sepsis Trusts describes sepsis screening as a two-part process, recommending that patients are ‘screened for sepsis’ if they have a NEWS2 score of 5 or more. It was difficult to determine whether the DSA described by respondents was the ‘prescreen’ to identify which patients needed screening for sepsis. Trusts which did not explicitly state that they used NEWS2 are highly likely to be using NEWS2 as part of their sepsis screening system, however, this may not be digital or not be considered part of the DSA. The combined paper and digital model requiring significant staff input to determine the requirement for review reduces some of the advantages of automation.

We opted to use an FOI request to increase response rate. People completing FOIs in NHS Trusts will not necessarily have the knowledge to answer the questions and err on the side of caution. Although some Trusts responded to the FOI with ‘not known’ or equivalent we are sure that there are staff in the Trust who know the algorithm being used. Finally, a minority of Trusts were unwilling or unable to give detailed information about their EPR or the underlying algorithm used for their sepsis alert. This is an important aspect of the introduction of digital alerts in hospitals in England. Currently, there is no necessity for hospitals to use approved digital alerts and no clear approval process. Two high-profile alerts have recently been identified as having no utility and at worst, causing patient harm.^14 26^

Wong *et al*^14^ have highlighted that in the USA ‘the ease of integration within the EPR and loose federal regulations’ means that hospitals adopt algorithms with ease, without a detailed knowledge of real-world performance. This is also the case in England, however, the Medical Health Regulatory Authority are now recommending that software as a medical device should undergo proper scrutiny, ‘commensurate with risk’. There is a need for a strong methodological library for evaluating digital tools, including determining risk. This is the focus of the UK NIHR DiAlS study that is evaluating electronic sepsis screening tools which are currently in use in England.

## Conclusion

Digital tools currently in use in acute hospitals in England use simple algorithms, based on paper-based T&T systems and are not taking advantage of granular data available in the EPR. While the majority of NHS Trusts in England are using NEWS2, as required in the National Standard Contract, this was not designed as a digital tool nor developed within data rich environments. Many Trusts are using alternative algorithms, often in combination with NEWS2, which do not have a strong evidence base. Studies which compare these approaches are vital to inform on the most effective practice.

As EPRs become universal, there is enormous potential in harnessing granular data to improve the performance of digital tools to support care of deteriorating patients. However, we need a strong methodological evaluation approach and clinicians and hospital leaders have a responsibility to understand the digital tools in use in their hospitals. We would go further and suggest that there should be a publicly accessible registry of digital alerting tools in use in hospitals, including DSAs.

## Data Availability

Data are available on reasonable request. Please contact the DiAlS Team for the information in a spreadsheet format. All data are publicly available on individual NHS Trust’s websites.
