# Supplementary material for: Prevalence of electronic screening for sepsis in National Health Service acute hospitals in England
Source: BMJ Health Care Inform. 2023 May 11;30(1):e100743. doi: 10.1136/bmjhci-2023-100743 (PMC10186434; doi:10.1136/bmjhci-2023-100743)
Supplement: Supplementary data [file bmjhci-2023-100743supp001.pdf]

Supplementary Materials – FOI request

To Whom it May Concern,

I am lead researcher for a project looking into digital sepsis alerts in England and Wales [https://www.imperial.ac.uk/school-public-health/primary-care-and-public-health/research/global-digital-health/research/dials/]. We are interested in which alerting algorithms are in use in different NHS Trusts.

As part of this we would like information on digital sepsis alerts in your Trust.

Could you provide the following information:

a) Does your Trust use an electronic health/patient record? YES/NO

If YES

b) Who is the provider of the electronic health/patient record?
eg EPIC and Cerner
If you use more than one system in the hospital could you provide details of the system used for adult inpatients and adult emergency departments.

c) When was the electronic health/patient record introduced?

d) Does your Trust use a digital sepsis alert[1]? YES/NO

If YES

e) Please give details on the hospital departments in which the alert is active.

f) Please provide details of the algorithm[2] and/or thresholds[3] in use in the digital sepsis alert . If different algorithms and/or thresholds are in use in different hospital departments, please provide this information.

[1] Digital sepsis alert: an electronic notification that the patient may be at risk of developing sepsis or may have sepsis. This may notify clinicians as a pop-up in the electronic health record, a flag next to the patient on a ward list or a message/bleep to a mobile phone or bleeper.

[2] Algorithm – this may be NEWS2 or Red Flag Sepsis; an algorithm provided by the EHR provider, such as the St John Sepsis Algorithm provided by Cerner; or a bespoke algorithm designed in house.

[3] Information on thresholds would include the level at which the clinical observation contributes to the alert, such as a lactate value of 2.0mmol/litre.
